# Measurement of Low Concentration of Micro-Plastics by Detection of Bioaffinity-Induced Particle Retention Using Surface Plasmon Resonance Biosensors

**DOI:** 10.3390/bios11070219

**Published:** 2021-07-03

**Authors:** Chen-Ji Huang, Gudivada Vijaya Narasimha, Yu-Cheng Chen, Jen-Kun Chen, Guo-Chung Dong

**Affiliations:** 1 Institute of Biomedical Engineering and Nanomedicine, National Health Research Institutes, Maoli 350, Taiwan; cjhuang@nhri.edu.tw (C.-J.H.); 044035@nhri.org.tw (G.V.N.); jkchen@nhri.edu.tw (J.-K.C.); 2 Department of Life Sciences, National Central University, Taoyuan 320, Taiwan; 3 National Institute of Environmental Health Sciences, National Health Research Institutes, Maoli 350, Taiwan; yucheng@nhri.edu.tw

**Keywords:** biosensor, surface plasmon resonance (SPR), micro-plastics, estrogen receptor

## Abstract

The issue of micro-plastics is becoming more and more important due to their ubiquity and the harm they cause to the human body. Therefore, evaluating the biological–physical interaction of micro-plastics with health cells has become the focus of many research efforts. This study focuses on the movement mode and low concentration detection development for micro-plastics in surface plasmon resonance (SPR). Firstly, 20-micrometer micro-plastics were prepared by grinding and filtering, and the movement mode was explored; then, the characteristics were investigated by SPR. Chromatographic analysis showed that the surface charge of micro-plastics dominated the elution time, and estrogen receptors (ERs) played a supporting role. A difference of micro-plastics in SPR sensorgram was observed, inferring the micro-plastics’ movement in rolling mode on the ERs. Characteristics analysis indicated that the low particle number of micro-plastics on SPR showed a linear relationship with the response unit (RU). When ERs were immobilized on the biosensor, the force of the binding of micro-plastics to ERs under an ultra-low background was equivalent to the dissociation rate constant shown as follows: PS (0.05 nM) > PVC (0.09 nM) > PE (0.14 nM). The ELISA-like magnetic beads experiment verified the specificity between ERs and micro-plastics. Therefore, by using the SPR technique, a biological-derived over-occupation of PS was found via higher binding force with ERs and longer retention time. In the future, there will be considerable potential for micro-plastics issues, such as identification in natural samples, biomarking, real-time detection in specific environments/regions and human health subject.

## 1. Introduction

Micro-plastics appear in daily life [1,2,3], but the subsequent waste causes trouble and difficulty in disposal. The presence of micro-plastics can be found in the Arctic and Alps [1], in the major oceans [4] and also in snowdrifts or rain, lake water, river water and soil [1,5,6,7,8]. Micro-plastics enter the human body through various paths, such as the skin, respiratory tract and digestive tract [9], and they are known from cell and animal experiments to cause much harm [2,10]. At this stage, chemical analyses are commonly used to confirm the micro-plastic species/type, such as Raman, IR, ATR/FTIR, etc. [11,12]. The detection limit for micro-plastics by a Raman microscope reaches 1 μm, while the detection limit of FTIR combined with an infrared microscope is 25 μm. However, if the micro-plastics exist in a liquid state, the instrument’s detection sensitivity will be disrupted due to the interference of environmental moisture. Even micro-plastics with an average particle size of 20 μm cannot be observed in a Raman spectrometer. Therefore, it is necessary to perform preprocessing procedures to separate the micro-plastics from water and sediment before detection [13,14]. This means that chemical analysis cannot be used to detect the particles in real time if the liquid samples are not pre-treated. 

Studies have reported methods of preparing micro-plastics by cutting and filtering and identifying them by microscope, GC/MS, Raman and FTIR [15,16]. In addition, it has been reported that micro-plastics can be deposited in organisms and gradually accumulate [9]. Dr. Hwang’s research pointed out that micro-plastics are a potential immune stimulant source inducing cytokines’ and chemokines’ production in a size-dependent manner [17]. In addition, Dr. Deng put forward a hypothesis about micro-plastics’ kinetics and distribution patterns in the liver, kidneys and intestines. Micro-plastics’ size is a significant factor. The distribution and concentration of larger molecular micro-plastics (20 μm) are more obvious compared to small molecules (5 μm) in the liver [18]. Therefore, developing a low-concentration real-time micro-plastic detection method will become an important matter for human health. 

Biosensors have been widely used for the qualitative and quantitative assessment of viruses and microorganisms [19,20]. The widely used enzyme-linked immunosorbent assay (ELISA) immunoassay uses estrogen receptors to specifically identify estrogen-like compounds [21,22]. Quartz crystal microbalance (QCM) can detect mass deposited on the surface sensor based on the oscillation shift of its fundamental and overtone resonance frequency as driven by current; at the same time, the particle size on the chip surface can also be obtained [23,24]. A field-effect transistor (FET) is a transistor that relies on an electric field to control the shape and conductivity of the “channel” in semiconductor material. An FET uses both biomolecule and receptor binding to change the surface charge distribution and surface potential for detection. Both QCM and FET have the characteristics of high sensitivity and being label-free, but an FET requires a lower volume for detection [25]. The current research focuses on biomolecules’ interaction between immobilizing on a biosensor and transporting in a flow channel (molecular level/molecular level). However, there is currently no discussion on the micro-sized particles aspect. At present, it is only known that Dr. Fang’s research used QCM combined with Raman spectroscopy and Fourier-transform infrared spectroscopy (FTIR) to conduct research on micro-plastics [26]. 

Surface plasmon resonance (SPR) is a photoelectric effect exited at metal-dielectric interface. and capable of monitoring the molecular interaction between in real time without labeling the reacts, and further providing equilibrium dissociation constants and detail kinetic in-formation by using analytic software. The SPR response (R) can be represent as following Langmuir equation. So effectively the starting value of free ligand sites (C) is converted to the maximum signal (R_max_) at saturation. The point is that the maximum signal depends on the size of the analyte and is generally reached with analyte concentration above 50 times *K_D_* (*K_D_* = *k_d_*/*k_a_*) of the interaction.

dR/dt = *k_a_*.C.(R_max_ − Rt) *k_d_*.Rt

The “interaction” concept of binding responses detected by SPR is with wide range of analytes including proteins (ligands, receptors, antibodies), nucleotides, small molecules, nanoparticles and even intact live cells. Therefore, SPR application can be further ex-pended to new drug discovery [27,28,29,30,31,32]. Many advantages of SPR in biomedical applications have been reported, such as label-free assay and real-time detection of nano-scale molecular interactions at very low concentrations (~pM) [33]. However, the interaction among micro-scale macromolecules is rarely mentioned compared to intermolecular behavior detection in aqueous conditions. A disparity in the binding force between hormone receptors and various micro-plastics has been pointed out [34,35,36]. Furthermore, few studies reported about real-time identification of low-concentration micro-plastics by SPR, especially for >10 μm particle size micro-plastics. 

**Figure 1:**
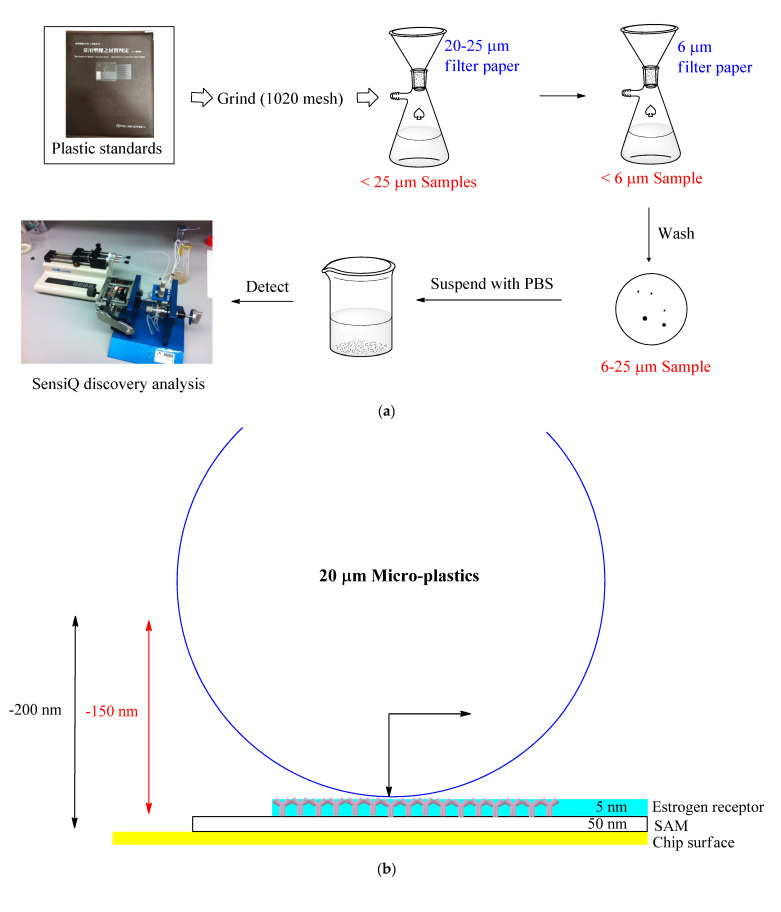
Schematic presentation of the experimental procedure. (**a**) General procedure of detection of micro-plastics based on SPR biosensor. (**b**) Schematic presentation of micro-plastic detection using ERs immobilized on COOH5 SPR sensor. Here, 200 nm (double arrow) refers to the detectable range of SPR; 150 nm (double arrow) refers to the detectable range of micro-plastics after subtracting the SAM and estrogen receptors immobilized on the chip; the arrow inside the particle indicates the driving force of gravity and the flow rate of the micro-plastics in the microfluid.

This study focuses on developing a low-concentration real-time micro-plastics detection system using estrogen receptors in SPR. Standard plastics were ground (1200 mesh) and filtered twice with filter paper (20–25 and 6 μm, respectively). The micro-plastics on the 6-micrometer filter paper were suspended with PBS and then detected by SPR (Figure 1a). Chromatography was used to analyze the interaction between the micro-plastics and ERs. In the specific micro-plastic identification process, estrogen receptor (ER) was immobilized on the biosensor by plasma, and then, the micro-plastics amount was measured using this biosensor in SPR (Figure 1b). The reaction mode and real-time detection of micro-plastics in SPR are also discussed. 

## 2. Materials and Methods

### 2.1. Preparation of 20-Micrometer Micro-Plastics 

As shown in Figure 1a, plastic standards (PE, PS and PVC) obtained from the Plastics Industry Development Center were ground with 1200 mesh aperture sandpaper. Particles large than 25 μm were removed on 20–25-micrometer filter paper by suction, and then, micro-plastics were obtained on 6-micrometer filter paper by suction. Micro-plastics of 20 μm in particle size on average (6–25 μm) were eluted with 2 mL PBS and collected in a glass sample bottle. 

### 2.2. Chromatography of Micro-Plastics 

A HisTrap HP (Cytiva, Uppsala, Sweden) column was installed for HPLC, reaching equilibrium with PBS at a flow rate of 25 μL/min. At the same flow rate, about 5 particles of micro-plastics were injected and analyzed during a 12-minute assaying period. Under the same conditions, 1.2 μg of ER was immobilized on resin, and then the interaction analysis with the micro-plastics was performed. 

### 2.3. Detection of Micro-Plastics on SPR 

Refer to Dr. Jani et al. for the quantitative method of PS latexes by SPR [37]. The SPR experiments were performed on a SensiQ discovery device (ICX Nomadics, Oklahoma City, OK, USA) at 25 °C with PBS (pH 7.4) as the running buffer. Instrument operations and data processing were carried out with the SensiQ discovery Control software and QdatTM analysis software, respectively. Without hormone receptors immobilized on the COOH5 sensor chip (ICX Nomadics, Oklahoma City, OK, USA), the 20-micrometer PE micro-plastic stocks were diluted to a concentration series spanning 10–120 particles in 30 μL. When the flow rate was 50 μL/min and a steady state was displayed, 30 μL of PE micro-plastics of different concentrations was injected to observe the relationship between the response and concentration of various PE micro-plastics on sensor chips. Each experiment was verified by three replicates. 

Human estrogen receptor alpha protein (300 ng, Abcam, Cambridge, UK) was immobilized on COOH5 sensor chips using plasma. Micro-plastic stocks were diluted to a concentration series spanning 2 particles in 80 μL according to the above quantitative method utilizing SPR. Once the flow rate of 50 μL/min showed a steady state, 80 μL of sample including micro-plastics was injected to observe the change in refraction angle. For the qualitative testing, each of the three 20-micrometer micro-plastic stocks was diluted to a concentration series spanning 10–100 particles in 30 μL. When the flow rate of 50 μL/min showed a steady state, 30 μL of micro-plastics of different concentrations/types was injected for observing the binding difference to ERs and the data were analyzed later (shown in Figure 1b). Each experiment was verified by three replicates. 

### 2.4. Magnetic Beads Verify the Interaction between ER and Micro-Plastics 

About 0.35 μg surface-modified Ni-NTA magnetic beads (30 μm average particle size, Cube Biotech, Monheim, Germany) were washed with PBS, and 0.3 μg ER (His tag in N-Terminus) was added and incubated for 60 min at 25 °C. Unreacted ERs were removed with PBS, and various concentrations of fluorescent PE micro-plastics (20 μm average particle size, Cospheric LLC, Santa Barbara, CA, USA) were added and reacted with the ERs for 60 min at 25 °C. After washing the unbound PE micro-plastics with PBS, the magnetic beads were removed using ENV-YEV protease for 18 h at 4 °C. Samples without magnetic beads (ER-PE micro-plastics) were taken and the fluorescence was measured according to the different concentrations of PE micro-plastics bound to ERs (SpectraMax^®^ Paradigm^®^ Molecular Devices, LLC, Santa Barbara, CA, USA) (Ex/Em: 575/607 nm).

## 3. Results

### 3.1. Preparation of 20-Micrometer Micro-Plastics

**Figure 2:**
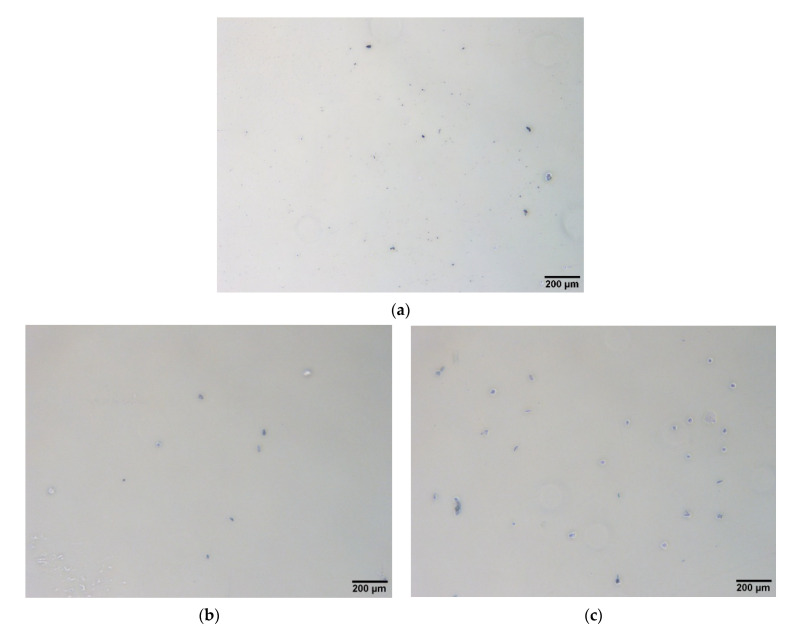
Observation of the prepared micro-plastics by a microscope. After grinding and filtering the plastic standard, micro-plastics with an average particle size of 20 μm were obtained; then, the morphology of these micro-plastics was observed using an inverted microscope. (**a**) PS, (**b**) PE and (**c**) PVC.

After treating the plastic standards by grinding and filtering twice with filter paper, the average 20-micrometer micro-plastics (6–25 μm) expected for subsequent experiments were obtained. We tried to observe the morphology with an inverted microscope, and ~20-micrometer particles could be observed, regardless of whether using PS, PE or PVC (Figure 2). Since these particles were prepared by grinding, they presented a variety of appearances, though all were basically micro-plastics. At the same time, we also observed particles larger than ~20 μm under the lens of the microscope. Most of these particles came from the solvent (deionized water)-eluted micro-plastics from the filter paper. 

### 3.2. Using Liquid Chromatography to Observe the Interaction between Micro-Plastics and Estrogen Receptors 

**Figure 3:**
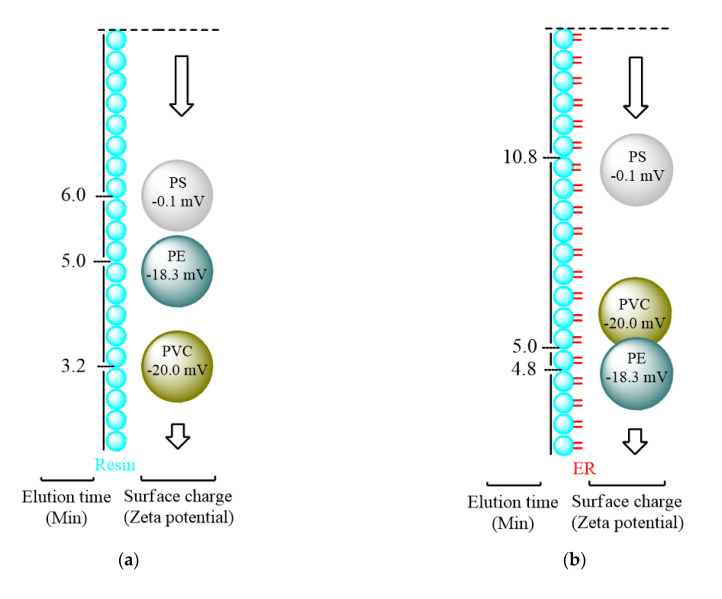

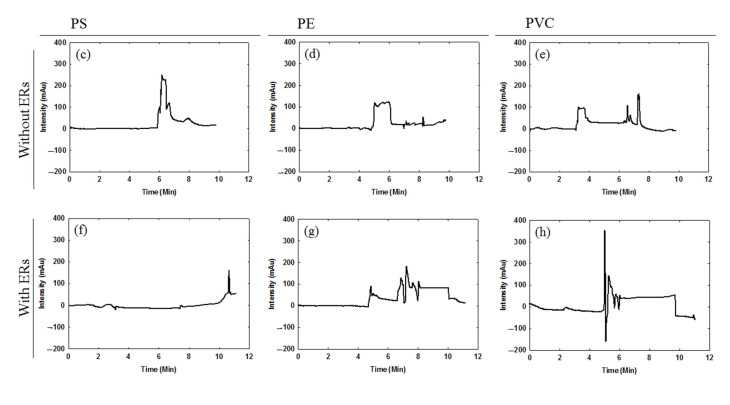
Validation of the interaction between micro-plastics and estrogen receptors by liquid chromatography. Schematic diagram of surface charge and elution time of micro-plastics without ERs (**a**) and with immobilized ERs (**b**) in liquid chromatography. In the absence of ERs, the micro-plastics interacted with Ni-NTA resins, and the measured elution time was (**c**) 6 min for PS, (**d**) 5 min for PE and (**e**) 3.2 min for PVC. After the ERs were immobilized on Ni-NTA resins, the micro-plastics interacted with the ERs and the measured elution times were (**f**) 10.8 min for PS, (**g**) 4.9 min for PE and (**h**) 5 min for PVC. The mobile phase was PBS at 25 μL/min including about 5 particles of micro-plastics.

Liquid chromatography was used to verify the binding of micro-plastics to ERs following the detection conditions of SPR. According to the surface charge characteristics, the elution time for different micro-plastics staying in resins was different (Figure 3a), and when the ERs were immobilized, the separation effect was enhanced (Figure 3b). PS, with the largest surface charge, stayed in resins for the longest time; on the other hand, PVC stayed in resins for the shortest time because of the smallest surface charge [29,30]. However, regardless of whether the surface charge was high or low, we could not clearly distinguish the five particles of micro-plastics during the detection period. Once the ERs were immobilized on Ni-NTA, it was found that PE did not extend the elution time due to ERs’ effects. However, in the same situation, the elution time of PVC was extended by about 1.8 min, and that of PS was increased by nearly 5 min compared to resins. Therefore, we inferred that the surface charge influence on micro-plastics is a major factor, and the ERs played a supporting role, making the effect of surface charge an additive effect (Figure 3c–h). Due to the small surface charge between PE and PVC, the identified effects were not enhanced even with ERs supplemented.

### 3.3. Micro-Plastics Movement Mode Speculation in SPR

**Figure 4:**
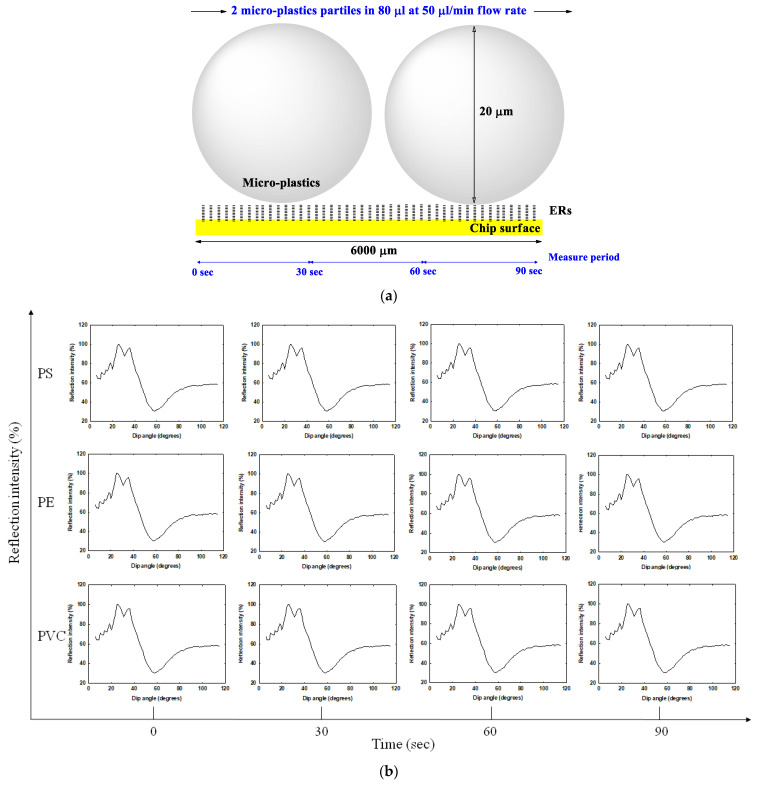

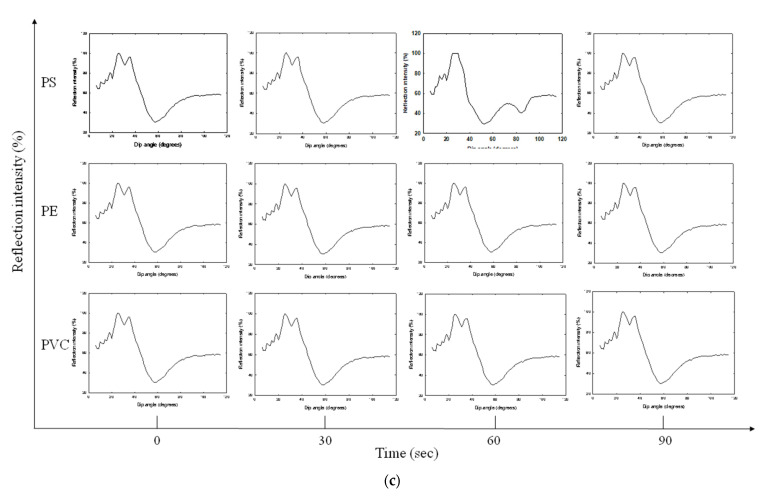
Refraction angle changes of microfluidic in SPR. (**a**) Schematic diagram of micro-plastics’ movement in microfluidic; PS, PE and PVC reacted without (**b**) or with (**c**) ERs resulting in a refraction angle change were recorded.

The detectable range of SPR was 200 nm; however, the particle size of the micro-plastics (average 20,000 nm) was much larger than this range. Therefore, we assessed the movement mode of the plastic particles in SPR. As shown in Figure 4a, the SPR microfluidic was about 6000 μm, and two plastic particles at most passed through at the same time. The past experimental value showed that if the reaction intensity (RU) was used as the detection unit, the 10 particles had reached the detection limit. Therefore, we decided to detect the refraction angle instead of the response intensity to detect single particles. Figure 4b shows the refraction angle change for different micro-plastics. We performed detection at 30, 60 and 90 s. The results show that PE and PVC did not demonstrate any changes; for PS, significant refraction angle changes at around 60 s (Figure 4b) were pointed out. This phenomenon was perhaps caused by PS being detected reacting with ERs around this time. Combined with Figure 3, we speculated micro-plastics movement in a rolling manner in SPR and interaction with ERs. 

### 3.4. Determination of Micro-Plastics Concentration by SPR

**Figure 5:**
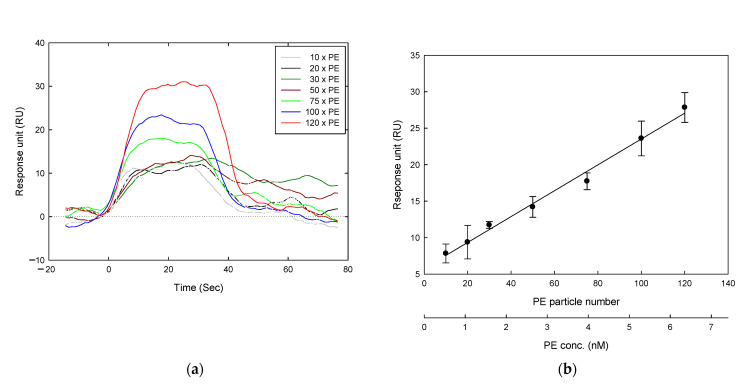
Calculation of micro-plastics concentration by SPR. When ERs were not immobilized on biosensors, various concentrations of PE micro-plastics flowed through, and the relationship between concentration and absorption was observed. (**a**) Raw data for micro-plastics and reaction intensity during reaction period. (**b**) The relationship between micro-plastics number/concentration and absorption was plotted; the absorption value was taken according to the point in (**a**). It can be seen that in the absence of estrogen receptors, the micro-plastics had a linear relationship with the relative absorption (response unit, RU). All data were repeated twice. Reaction volume: 30 μL; reaction flow rate: 50 μL/min.

In the absence of estrogen receptors, the relationship between PE micro-plastic numbers and response intensity was observed utilizing SPR. Figure 5a shows that the response intensity increased proportionally to the PE micro-plastic numbers after deducting the background. Analyzing the PE micro-plastics and the corresponding response intensity (the average value of the highest absorption area shown in Figure 5a), we found that the particle number was positively correlated with the response intensity (Figure 5b). We then converted the plastic numbers into an equivalent concentration, which also followed the correlation described above. Therefore, SPR can be used as a powerful tool for quantifying micro-plastics. 

### 3.5. Measurement of the Interaction between ER and Micro-Plastics by SPR

**Figure 6:**
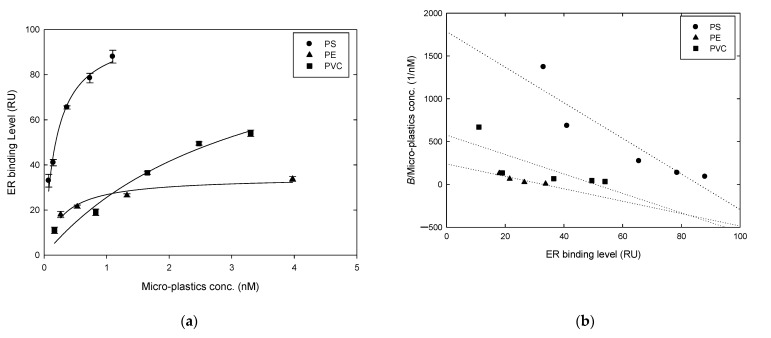
Specificity test for micro-plastics to estrogen receptors by SPR. Various concentrations of micro-plastics reacted with the same concentration of ERs immobilized on the chip, and SPR was utilized to observe the relationship between micro-plastics’ concentration and binding level of ER. (**a**) The Langmuir equation was used to plot the binding level of micro-plastics to ERs, showing that the three micro-plastics each had a different affinity for the ERs. (**b**) Plot according to (**a**) of the obtained ER binding level, bound amounts and micro-plastics’ concentration at which the dissociation constant was obtained. All data were repeated twice; (**b**) was calculated with numerical averages. Reaction volume: 30 μL; reaction flow rate: 50 μL/min.

After quantifying the micro-plastics according to Figure 5, the next stage was to perform real-time low-concentration qualitative micro-plastic determination. Under the ultra-low background values premise, the experimental results in the presence of estrogen receptors are shown in Figure 6 for the qualitative determination of three different micro-plastics. Different concentrations of the micro-plastics acted on the same immobilized amount of ERs (approximately 9000 RU immobilized on the biochip), and SPR was used to observed the relationship between the binding level and concentration. The Langmuir equation [38,39] was used to derive the maximum binding capacity (*B_max_*) and the equilibrium dissociation constant (*K_D_*) values of PS, PE and PVC (Figure 6a):

(1) $$Bound\;\left(B\right)=\frac{B_{Max}\times X_{Micro-plastics\;conc}}{K_{D}+X_{Micro-plastics\;conc}}$$

We plotted the ratio of the ER binding level (*B*) to the micro-plastic concentration ($\frac{B}{X_{Micro-plastics\;conc}}$) and the relative ER binding level (*B*), and the calculated slope was −1/*k_d_* (Figure 6b).

(2) $$B\times X_{Micro-plastics\;conc}+B\times K_{D}=X_{Micro-plastics\;conc}\times B_{Max}$$

(3) $$\frac{B}{X_{Micro-plastics\;conc}}=-\frac{1}{K_{D}}\times B+\frac{B_{Max}}{K_{D}}$$

If measuring the molecular type of small molecules as the detection mode and assuming that the micro-plastics’ morphology was round, the *K_D_* value was calculated from high to low in the following order: PS (0.19 nM) > PE (0.29 nM) > PVC (3.32 nM) (Table 1). However, the micro-plastics’ size was much larger than the molecular weight (micron vs. nanometer), and the detection process was similar to that of the micro-plastics “intercepted” by many estrogen receptors in a rolling state. Therefore, the dissociation rate constant (*k_d_*) was considered, which fit more with the actual state. The greater the *k_d_* value is, the greater the resistance is. This indicated that the micro-plastics with a lower affinity to ER have higher *k_d_* values. Therefore, the affinity to ER from high to low was PS (0.05 nM) > PVC (0.09 nM) > PE (0.14 nM). This difference in different micro-plastics’ specificity in acting on estrogen receptors has the potential for use in the real-time low-concentration detection of micron-sized micro-plastics in the future. 

In addition, as estrogen has a very high affinity for ERs, the *K_D_* value could not be detected based on the current experimental parameters (reaction volume: 30 μL; reaction flow rate: 50 μL/min). Di(2-ethylhexyl) phthalate (DEHP) was used as a standard substrate to assay the relationship to ER affinity (Table 1). We observed that under the same experimental conditions, macromolecular particles (micro-plastics) and small molecular particles (DEHP) belonged to different stages in either affinity equilibrium state (referring to the K_D_ value) or dissociation state (referring to the *k_d_* value). 

### 3.6. Verification of the Interaction of Micro-Plastics on Estrogen Receptors by ELISA

**Figure 7:**
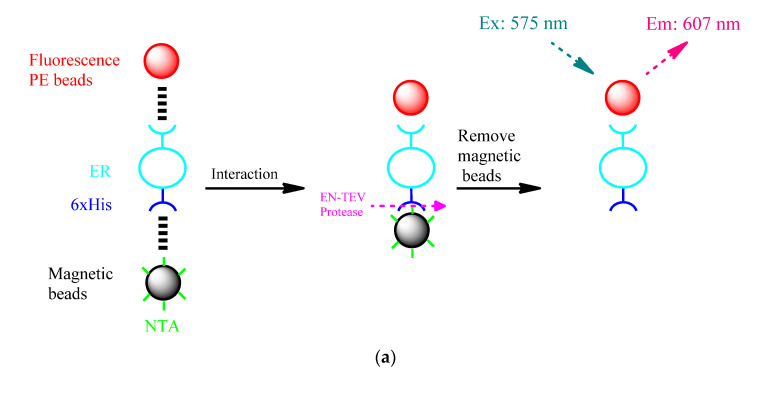

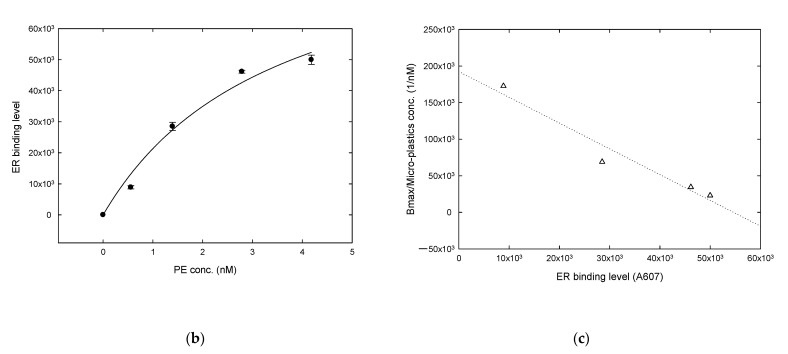
ELISA-like method for determining PE micro-plastics. (**a**) General procedure showing the detection process. The estrogen receptor was immobilized on magnetic beads’ surface; then, the PE-fluorescence micro-plastics were used to interact with the ER. Magnetic beads were removed after cutting with EN-PEV protease, and the difference in fluorescence values was observed (Ex/Em: 575/607 nm). (**b**) The PE micro-plastic concentration and fluorescence absorption value based on Langmuir were plotted, which indicated the relationship between PE micro-plastics and the ER binding level. (**c**) Plotted according to (**b**), the obtained ER binding level, bound amounts and micro-plastics concentration at which the dissociation constant was obtained. All data were repeated twice ((**b**) was calculated with numerical averages).

A model similar to ELISA was used to further verify the specific effect of micro-plastics on ER. As shown in Figure 7a, ERs were specifically bound on magnetic beads’ surface, which is similar in size to micro-plastics (~30 μm); then, the ERs were interacted with PE-fluorescence micro-plastics. In order to eliminate the shielding effect from the magnetic beads, the EN-TEV protease recognized and cleaved at Gly/Ser in Glu-Asn-Leu-Tyr-Phe-Gln-(Gly/Ser) sequence on 6xHis after the PE-fluorescence micro-plastics’ finished reacting with the ERs. ER–PE-micro-plastics complexes were observed by detecting the fluorescence from PE beads at 607 nm. In the control group experiment with only fluorescent PE micro-plastics (without ERs), fluorescence value is close to the background value, as shown in Figure 7b, the fluorescence value of ERs-PE micro-plastics complex also followed Langmuir equation. The *K_D_* and *k_d_* calculated as 3.51 and 0.28 nM, respectively (Figure 7c, Table 1), which verified the specific interaction between micro-plastics and ERs, further. In addition, the methodology between SPR and ELISA was compared found that the ER had lower binding level on ELISA mode at the same ERs immobilization concentration which means the surface area of spherical magnetic beads for immobilizing ERs was smaller than the SPR’s. On the other hand, as the ELISA-magnetic bead mode was static that was easier to “intercept” PE micro-plastics and resulting a larger *k_d_* value. 

## 4. Discussion

In this study, we used SPR to explore the movement patterns and detection methods of micron-sized plastics and performed verification via liquid chromatography and ELISA. The simple and clear grinding and filtration methods were used to prepare micro-plastics with an average particle size of 20 μm, which were then detected by SPR based on its low concentration sample detection characteristic. The main factor behind ultra-low-concentration particles being retarded in the chromatographic column was the surface charge of the micro-plastics themselves, and the ERs enhanced the surface charge effects. Therefore, PS further extends the retention time in the presence of ERs. SPR refraction angle changes were observed in PS samples under the same conditions. In addition to echoing the HPLC results, this result also verified that large-sized particles (micro-graded micro-plastics vs. nano-level ERs) interacted with ERs in a rolling manner in SPR microfluidics. The Langmuir equation was used to analyze micro-plastic adsorption kinetics by Dr. Guibal [40]; we also used the same equation for exploring the binding force between micro-plastics and ERs. As shown in Figure 4, it was deduced that the micro-plastics interacted with ERs in a rolling mode in microfluidics. The dissociation constant (*k_d_*) analysis was more appropriate and fit the real status better than the equilibrium dissociation constant (*K_D_*) did. SPR data were calculated for the binding force (*k_d_*) for ERs from high to low, in the order of PS (0.05 nM) > PVC (0.09 nM) > PE (0.14 nM), showing that PS had the highest binding force for ERs, and the *k_d_* value was relatively the smallest. PE had the lowest binding force to ERs, and the *k_d_* value was the largest (Table 1). The *k_d_* values of the three micro-plastics were also consistent with the retention time results in HPLC: PS, with the smallest *k_d_* value (highest binding force), had the longest retention time; PE, with the smallest binding force, had the shortest retention time (Figure 3). In addition, the data showed that ERs had the best binding effect to PS, so they could serve as a potential biomarker for real-time low-concentration PS detection in SPR. 

## 5. Conclusions

Nowadays, three of methods are used generally to detect micro-plastics. Among these, both of the hot needle test and fluorescent dyeing method are commonly used to quantify, but not to qualify micro-plastics. Another method is micro-spectroscopy method. The method can both quantify and qualify micro-plastics. However, micro-spectroscopy method need an expensive equipment and complex sample pre-treatment to reduce interferences. So that, the method cannot be utilized to obtained real-time data. Otherwise, in micro-spectroscopy method the light source beam width is large. This is because they have low resolution in detectable plastic size and particle dimeter >100 μm. In this ER/SPR system, 20 μm of particle size was quantitatively detected. We built up micro-plastics preparation by grinding and filtering for an average of 20 μm particle size. Additionally, different plastics could be qualitatively identified dependent to their binding affinity to ER and particle motion. The quantitative analysis and qualitative detection for low-concentration micro-plastics was progressing via SPR. This make it possible to detect signal particle. In the future, research could be conducted on issues such as qualitative assessment of micro-plastics in natural samples, establishment of micro-plastic biomarkers for real-time detection in specific environments/regions and human health. 

## Figures and Tables

**Table 1:** Affinity of estrogen-like compounds to estrogen receptor.

Compound	PS	PE	PVC	PE/Magnetic Beads	DEHP
*B_max_*	100.41	34.65	110.57	96,243.94	79.52
*K_D_* (nM)	0.19	0.29	3.32	3.51	1921.07
*k_d_* (nM)	0.05	0.14	0.09	0.28	1000.00

## Data Availability

All data relevant to the study are included in the article.
